# Efficacy evaluation of ultrasound-derived fat fraction in predicting of the severity of women with polycystic ovary syndrome and obesity

**DOI:** 10.3389/fmed.2026.1779305

**Published:** 2026-03-10

**Authors:** LingZhi Meng, JinXia Wang, WenJing Liu, Yue Qin, Zongli Yang, Yan Xu

**Affiliations:** 1 Department of Obstetrics and Gynecology Ultrasound, Qingdao Women and Children’s Hospital, Qingdao, China; 2 Department of Ultrasound, Gaomi People’ Hospital, Weifang, China; 3 Department of Abdominal Ultrasound, The Affiliated Hospital of Qingdao University, Qingdao, China

**Keywords:** hepatic steatosis, obesity, ovarian hemodynamics, polycystic ovary syndrome, ultrasound-derived fat fraction

## Abstract

**Introduction:**

To investigate the feasibility of ultrasound-derived fat fraction (UDFF) as an evaluation index for obese women with polycystic ovary syndrome (PCOS).

**Methods:**

A total of 103 Women with PCOS and obesity (case group) and 108 Women without PCOS but with obesity (control group) were enrolled. All participants underwent transvaginal three-dimensional ultrasound examination on the 7th day of their menstrual cycle. General data, biochemical parameters, sex hormones, ovarian volume, follicle count, ultrasound blood flow indices, hepatic UDFF, and shear wave velocity (SWV) were collected from both groups. Comparisons were made between the case group and the control group regarding UDFF, SWV, ovarian morphological indices, ovarian stromal blood flow parameters, clinical characteristics, serum biochemical indices, and sex hormone levels. Logistic regression analysis was used to identify risk factors for the case group, and Spearman correlation analysis was performed to assess the correlations between UDFF and various variables.

**Results:**

Compared with the control group, Women with PCOS and obesity had significantly higher values of the UDFF, Quantity of follicle, Luteinizing Hormone (LH), LH/FSH ratio, Estradiol (E2), Peak Systolic Velocity (PSV) of ovarian stromal arteries, End Diastolic Velocity (EDV) of ovarian stromal arteries, vascularization index (VI),(Vascularity Index, VI: Reflecting the richness of blood vessels within the detection area, it is a core parameter for assessing the level of angiogenesis using power Doppler ultrasound;) blood flow index (FI),(Flow Index, FI: It reflects the average intensity of blood flow signals within the detection area and has a certain correlation with blood flow velocity and volume;)vascularization-flow index (VFI),(Vascular Flow Index, VFI: By combining the comprehensive indicators of VI and FI, the vascular richness and blood perfusion intensity of the detection area can be comprehensively evaluated) Low-Density Lipoprotein (LDL), abdominal circumference, Triglyceride (TG), fasting blood glucose and Homeostatic model assessment-insulin resistance (HOMA-IR) (all *P* < 0.05). In contrast, Women with PCOS and obesity showed significantly lower SWV, Follicle-Stimulating Hormone (FSH),Resistance Index (RI),(Resistance Index, RI: The calculation formula is (systolic peak flow velocity—end diastolic flow velocity)/systolic peak flow velocity;) Pulsatility Index (PI)(Pulsatility Index, PI: The calculation formula is (systolic peak velocity—end diastolic velocity)/mean velocity.) (all *P* < 0.05). Logistic regression analysis revealed that SWV, VFI, UDFF, RI, and LDL were independent risk factors for Women with PCOS and obesity. The ROC curve showed that the area under the curve (AUC) of the multivariate model was 0.913. Spearman correlation analysis indicated that UDFF was correlated with SWV, ovarian morphological indices, ovarian stromal blood flow parameters, clinical characteristics, serum biochemical indices, and sex hormone levels.

**Discussion:**

Women with PCOS and obesity have higher UDFF values than the control group. UDFF is correlated with glucose-lipid metabolism, sex hormone indices, ovarian morphology, and hemodynamic parameters, suggesting that UDFF can predict the severity of PCOS to a certain extent.

## 1 Introduction

PCOS is the most common endocrine disorder in women, affecting 6–20% of women of reproductive age (1). It is associated with hyperandrogenism, metabolic changes, and reproductive abnormalities (2). PCOS can lead to various metabolic disorders. Studies have shown that most PCOS patients are complicated by insulin resistance (IR), hyperandrogenism (HA), and metabolic syndrome (Mets) (3). Women with PCOS and obesity have a higher probability of insulin resistance, which stimulates pancreatic β-cells to secrete large amounts of insulin, subsequently affecting sexhormone levels and ovarian ovulation function (4).Specific characteristics of lipid metabolism disorders in PCOS patients typically include elevated TG, decreased HDL, increased LDL with smaller particle size, and an imbalanced apolipoprotein A-I/B ratio. Abnormal lipid metabolism is a core feature and key comorbidity of PCOS (5). PCOS and glucose-lipid metabolism interact through mechanisms such as insulin resistance, obesity, hyperandrogenism, and chronic inflammation, forming a complex network (6). There is a negative correlation between ovarian blood flow parameters and LH and E2 levels, reflecting the influence of hormonal changes on ovarian morphology and follicle development (7). Studies have shown (8) that ovarian stromal artery VI, FI, and VFI values are higher in PCOS patients compared to the control group, while there is no statistically significant difference in RI and PI values between the two groups. Therefore, changes in hormones, glucose-lipid metabolism indicators, ovarian morphology, and blood flow parameters have clinical value as indicators for assessing PCOS condition.

UDFF is a novel technology for the quantitative assessment of hepatic fat content. It rapidly analyzes data on attenuation coefficients and backscatter coefficients integrated into ultrasound systems to obtain hepatic fat content measured as a percentage (9). Compared with BMI (a marker of overall obesity), UDFF more intuitively and accurately reflects hepatic fat deposition. Previous studies have demonstrated the potential of UDFF in diagnosing and grading hepatic steatosis, and its diagnostic value for hepatic steatosis in children, obese populations, and other groups has been confirmed (10), but few studies have focused on Women with PCOS and obesity. Previous studies have demonstrated that UDFF exhibits good diagnostic performance in grading mild, moderate, and severe MAFLD among women with PCOS and obesity, with corresponding cut-off values established. By investigating the correlation between UDFF and glucolipid metabolic indicators, sex hormone levels, ovarian morphological parameters, and ovarian hemodynamic indices in women with PCOS and obesity, this study provides a more targeted research entry point and data support for further exploration of the intrinsic relationship and mechanism between hepatic metabolism and ovarian metabolism. The Acuson Sequoia ultrasound diagnostic system can simultaneously measure UDFF and Automatic point shear wave elastography (auto-pSWE). Auto-pSWE is an innovative real-time two-dimensional elastography technology that can quickly and quantitatively assess liver stiffness. Studies have proven a positive correlation between tissue stiffness and shear wave propagation velocity—shear waves travel faster in stiffer tissues and slower in softer tissues (11). Previous studies have evaluated the accuracy of pSWE in quantitatively assessing the degree of hepatic steatosis in patients with Metabolic Associated Fatty Liver Disease (MAFLD) (12), confirming that pSWE has good diagnostic performance for hepatic steatosis in MAFLD patients.

This study focuses on women with PCOS and obesity, combining UDFF, auto-pSWE with hormone levels, glucose-lipid metabolism indices, ovarian morphology, and blood flowparameters to jointly manage women with PCOS and obesity, aiming to confirm that UDFF has good correlations with various indicators in women with PCOS and obesity.

## 2 Materials and methods

### 2.1 Study subjects

This was a single-center retrospective analysis based on a prospective cohort database, enrolling 103 obese women with PCOS who visited the Affiliated Hospital of Qingdao University from January 2025 to February 2026, and 108 age- and BMI-matched obese women without PCOS as the control group. Obesity was defined as BMI ≥ 28.0 kg/m^2^ according to the Chinese Guidelines for the Prevention and Control of Overweight and Obesity in Adults (13).

Inclusion criteria for the case group:

(1)    Premenopausal women over 18 years old with a first diagnosis of PCOS and BMI ≥ 28 kg/m^2^.
(2)    PCOS diagnosis was based on the 2003 Rotterdam diagnostic criteria (14).

Inclusion criteria for control group:

(1)    Premenopausal women aged > 18 years;
(2)    BMI ≥ 28 kg/m^2^;
(3)    No PCOS.

Exclusion criteria:

(1)    Patients with autoimmune liver disease, alcoholic liver disease, genetic metabolic liver disease, drug-induced liver injury, hepatic vascular disease, or liver tumors;
(2)    Patients with other confirmed endocrine disorders, such as hyperprolactinemia, thyroid disease, congenital adrenal hyperplasia, Cushing’s syndrome;
(3)    Patients with malignant tumors;
(4)    Patients who received sex hormone therapy within 3 months;
(5)    Patients with a history of pregnancy or lactation within 6 months.

### 2.2 Clinical data and laboratory examinations

Patient age, height, and weight were collected, and BMI was calculated using the formula: BMI = weight (kg)/height^2^ (m^2^). Total Cholesterol (TC) and Triglyceride (TG) levels were measured using deionization and enzymatic methods; Low-Density Lipoprotein Cholesterol (LDL-C) and High-Density Lipoprotein Cholesterol (HDL-C) levels were measured using chemically modified enzymatic methods; Fasting Blood Glucose(FBG) levels were measured using the hexokinase/glucose-6-phosphate dehydrogenase method; Fasting Insulin (FIns) levels were measured using chemiluminescence (double-antibody sandwich) method; Homeostasis Model Assessment-Insulin Resistance (HOMA-IR) was calculated using the formula: HOMA-IR = FBG (mmol/L) × FIns(mU/L)/22.5; Testosterone(T), Luteinizing Hormone (LH), Follicle-Stimulating Hormone (FSH), Prolactin (PRL), and Estradiol (E2) levels were evaluated using electrochemiluminescence immunoassay.

### 2.3 Ultrasound examinations

A Doppler ultrasound diagnostic system equipped with an endocavitary probe with a frequency of 5.0∼7.5 MHz was used. On the 7th day of the patient’s menstrual cycle, ovarian volume and follicle diameter were measured. After switching to Doppler blood flow imaging mode, PI, PSV, EDV, VI, FI, VFI, and other parameters of ovarian stromal arteries were carefully detected. Each parameter was measured repeatedly 3 times, and the average value was taken as the final result.

Measurements of hepatic UDFF and SWV were independently performed by two physicians with more than 5 years of clinical ultrasound experience. Equipment calibration was consistent, and the acquisition depth was 4.5∼5.5 cm from the liver capsule. Both ultrasound physicians were unaware of the patient’s clinical data. In case of discrepancies in ultrasound conclusions, a third physician with more than 20 years of clinical ultrasound experience was consulted for a decision.

Examination methods:

1. Patient preparation and position: Fasting for at least 4 hours, supine position, with the right arm placed near the head.

2. Sampling method: The probe was placed intercostally; UDFF and auto-pSWE software were activated. The depth marking line of the Region of Interest (ROI) was placed on the liver capsule, and the ROI was kept perpendicular to the liver capsule. Segment V of the liver was selected, avoiding large blood vessels, hepatobiliary ducts, and rib shadow areas. During acquisition, the patient held their breath under quiet breathing; 15 SWV and 15 UDFF values were measured by a single acquisition, and their average value, Standard Deviation (SD), median, Interquartile Range (IQR), and IQR/median were listed on the report page. According to the recommendations of the Chinese expert consensus (15), this study adopted the median value obtained from three measurements as the evaluation criterion for auto-pSWE and UDFF. The examiner performing UDFF measurements was blinded to the patient’s group assignment and clinical information. The interval between ultrasound-related examinations and laboratory tests did not exceed one week.

### 2.4 Statistical methods

R studio and GraphPad Prism 9.5.0 were used for data analysis and graph plotting. Sample size was calculated with two-tailed α = 0.05, power = 0.90, expected AUC = 0.90, null hypothesis AUC = 0.60 and case-control ratio = 1:1, yielding a theoretical total sample size of 42 cases. Shapiro-Wilk test was used for normality assessment. Normally distributed data were expressed as mean ± standard deviation and compared by independent samples *t*-test; non-normally distributed data as M(P25, P75) and compared by Mann-Whitney U test. BMI was corrected by non-parametric test. Taking PCOS status as the dependent variable (yes = 1, no = 0), indices with significant differences in non-parametric ANCOVA were standardized for univariate logistic regression; the top 5 variables with the highest AUC were selected for multicollinearity test (variance inflation factor VIF, tolerance) and stepwise logistic regression to identify independent predictors. ROC curves were plotted to calculate AUC, and Nagelkerke R^2^ was used to evaluate model fitting. Spearman correlation analysis was used to explore correlations between UDFF and other variables.

## 3 Results

### 3.1 Screening process of study subjects

**FIGURE 1:**
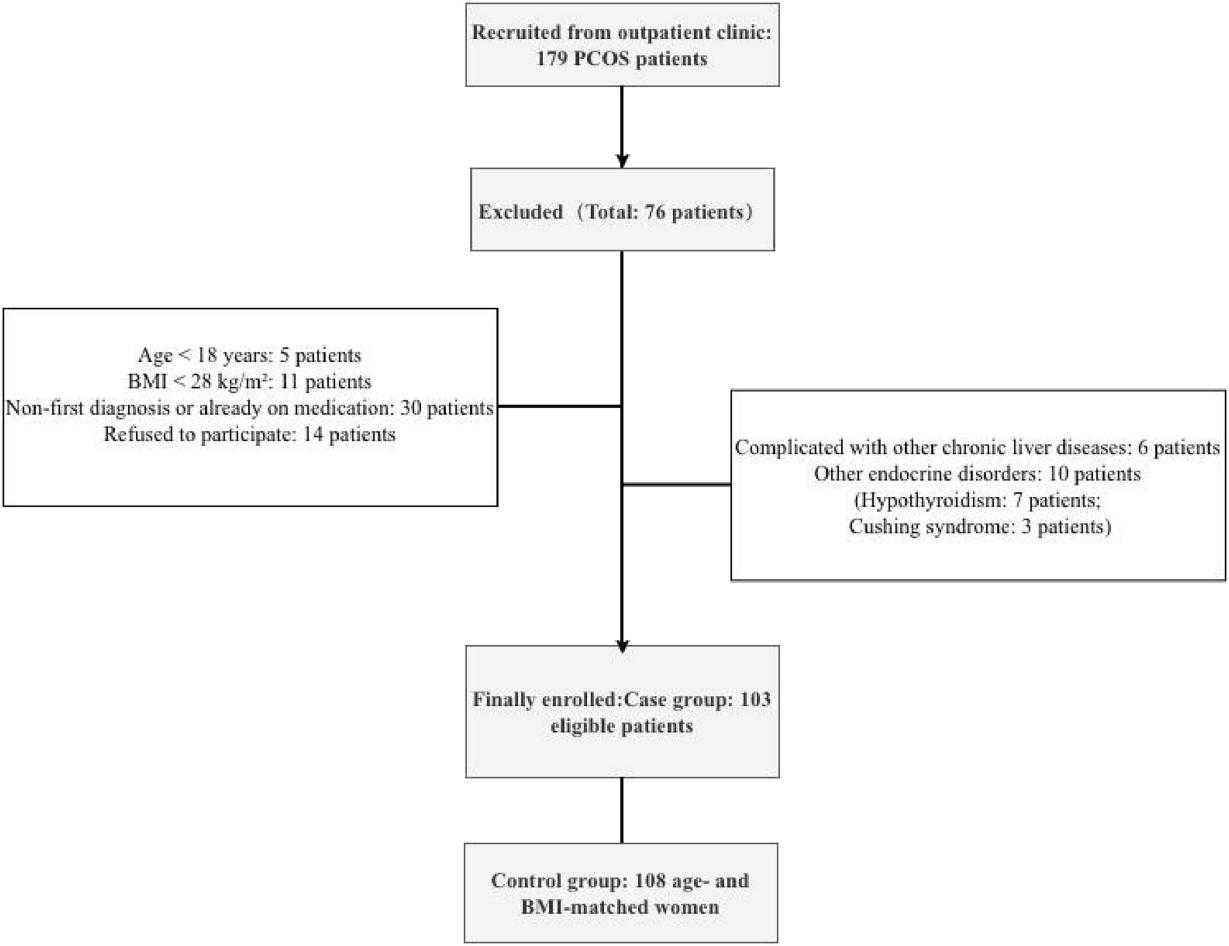
Participant flow diagram of a case-control study including women with obesity and PCOS and women with obesity but without PCOS.

A total of 179 patients with PCOS were recruited from the outpatient clinic. During the screening process, 76 patients were excluded for specific reasons: 5 patients were under 18 years of age; 11 patients had a BMI below 28 kg/m^2^; 6 patients with PCOS had other chronic liver diseases; 10 patients had other endocrine disorders (7 with hypothyroidism and 3 with Cushing’s syndrome); 30 patients with PCOS were not newly diagnosed or had already received medication; and 14 patients declined to participate. Finally, 103 eligible patients were enrolled in the case group, and 108 age- and BMI-matched women were included in the control group (Figure 1).

### 3.2 Clinical and ultrasound examination results

Compared with the control group, the case group had significantly higher quantity of follicle, LH, LH/FSH ratio, UDFF, VI, VFI, FI, EDV, PSV, E2, LDL, abdominal circumference, TG and fasting blood glucose (all *P* < 0.05), and significantly lower SWV, RI, PI, FSH and BMI (all *P* < 0.05). No significant intergroup differences were found in age, fasting insulin, IR, HDL or PRL (all *P* > 0.05) (Table 1).

**TABLE 1:** Comparison of general data between the two groups.

Variable	Women with PCOS and obesity(*n* = 103)	Women without PCOS but with obesity(*n* = 108)	*t*/*u*-value	*P-*value
Age(years)	25(23, 27)	25.00(24, 28)	5696.5	0.7611
BMI(kg/m^2^)	28.93(28.33, 31.25)	30.09(28.55, 31.52)	6646.5	0.0145
Abdominal Circumference(cm)	79.00(73.00, 92.50)	85.00(80.00, 92.00)	6561	< 0.001
LH(mIU/mL)	14.11(11.25, 19.88)	4.96(3.56, 7.67)	13.5	< 0.001
FSH(mIU/mL)	4.58(4.11, 5.33)	8.71(5.77, 11.08)	9061	< 0.001
LH/FSH	2.77(2.51, 3.44)	0.68(0.39, 0.87)	4166.5	< 0.001
T(ng/dL)	92.00(83.00, 165.00)	58.00(52.75, 79.00)	1486.5	< 0.001
E2(pg/mL)	264.00(230.50, 289.00)	189.00(157.50, 207.00)	1180	< 0.001
PRL(ng/mL)	322.00(238.00, 417.00)	311.00(282.50, 349.25)	4813	0.0913
TG(mmol/L)	3.68(1.46, 4.19)	1.77(1.60, 2.25)	3964.5	< 0.001
LDL	2.74(1.88, 3.55)	1.87(1.66, 2.33)	3083.5	< 0.001
HDL(mmol/L)	1.23(0.83, 1.48)	1.28(1.16, 1.41)	6255.5	0.1179
Glucose(mmol/L)	5.40(5.20, 5.80)	5.20(5.10, 5.40)	3800.5	< 0.001
Insulin(mU/L)	12.83 ± 6.70	11.64 ± 5.03	-1.4602	0.1459
IR	3.33(1.68, 4.31)	2.47(1.67, 3.66)	2880	0.0577
Quantity of follicle	13.00(12.00, 15.00)	9.00(7.75, 10.00)	714.5	< 0.001
VI(%)	7.93(6.12, 9.40)	5.32(5.01, 6.20)	1973.5	< 0.001
FI	36.03(32.68, 38.98)	30.34(30.00, 33.99)	2286	<0.001
VFI	2.91(2.01, 3.71)	1.61(1.50, 2.11)	2074.5	< 0.001
PSV(cm/s)	12.11(10.76, 15.66)	11.23(10.11, 13.44)	3831.5	< 0.001
EDV(cm/s)	4.51(4.01, 6.29)	3.66(3.02, 4.35)	2972	< 0.001
RI	0.63(0.60, 0.65)	0.68(0.64, 0.71)	8417	<0.001
PI	0.90 ± 0.12	1.01 ± 0.13	6.2725	< 0.001
SWV(m/s)	1.11(1.00, 1.21)	1.25(1.18, 1.28)	8683	< 0.001
UDFF(%)	10.00(7.00, 16.00)	5.00(4.00, 7.00)	2071.5	< 0.001

Although BMI matching was considered in the selection of the control group for this study, the matching criterion was uniformly set at BMI ≥ 28, without further refined stratification or matching of BMI values within this range. This ultimately led to a statistically significant difference in BMI between the two groups. Therefore, non-parametric ANCOVA was employed for adjustment. After correction, the difference between groups changed from non-significant to statistically significant for the following indicator: IR (Table 2).

**TABLE 2:** Non-parametric ANCOVA for BMI correction.

Variable	*F-*value	*P-*value (BMI-corrected)
Age(years)	0.093	0.7602
Abdominal Circumference(cm)	8.228	0.0046
LH(mIU/mL)	614.644	< 0.001
FSH(mIU/mL)	87.571	< 0.001
LH/FSH	603.413	< 0.001
T(ng/dL)	140.476	< 0.001
E2(pg/mL)	190.436	< 0.001
PRL(ng/mL)	2.901	0.09
TG(mmol/L)	14.931	< 0.001
LDL	40.418	< 0.001
HDL(mmol/L)	3.269	0.072
Glucose(mmol/L)	19.223	< 0.001
Insulin(mU/L)	1.999	0.1589
IR	4.327	0.0387
Quantity of follicle	282.05	< 0.001
VI(%)	98.627	< 0.001
FI	76.72	<0.001
VFI	90.829	< 0.001
PSV(cm/s)	16.746	< 0.001
EDV(cm/s)	41.453	< 0.001
RI	52.719	<0.001
PI	52.368	< 0.001
SWV(m/s)	65.721	< 0.001
UDFF(%)	91.666	< 0.001

### 3.3 PCOS Classification in the case group

Among the 103 obese women with PCOS, 78 (75.73%) had ovulatory disorders; 80 (77.67%) had hyperandrogenism(25 with clinical hyperandrogenism only, 2 with biochemical hyperandrogenism only, 53 with combined clinical and biochemical hyperandrogenism); 10 (9.71%) had polycystic ovarian morphology (PCOM); the main clinical phenotype was Phenotype 2 (ovulatory disorder + hyperandrogenism, 48.54%) (Table 3).

**TABLE 3:** PCOS classification in the case group (*n* = 103).

Baseline classification indicators	Specific indicators	Case count (n))	
Menstrual cycle	Ovulatory disorder	78	Cycle > 35 d, oligovulation (< 8 times/year) or amenorrhea (≥ 6 months)
Hyperandrogenism	Clinical only	25	Modified Ferriman-Gallwey score ≥ 8 or moderate-severe acne
	Biochemical only	2	Serum total T ≥ 75 ng/dL or free T ≥ 15 dg/mL
	Combined clinical & biochemical	53	Meeting both above criteria
	None	38	No abnormal clinical/biochemical indices
Polycystic Ovarian Morphology(PCOM)	Positive	10	≥ 12 follicles (2∼9 mm) in one/both ovaries, and/or ovarian volume ≥10 mL
	Negative	85	Not meeting PCOM criteria
PCOS Clinical Phenotypes According to the Rotterdam Criteria	Phenotype 1 (Ovulatory disorder + hyperandrogenism + PCOM)	18	Classic phenotype
	Phenotype 2 (Ovulatory disorder + hyperandrogenism)	50	No PCOM
	Phenotype 3 (Ovulatory disorder + PCOM)	18	No hyperandrogenism
	Phenotype 4 (Hyperandrogenism + PCOM)	10	Normal ovulation

### 3.4 Logistic regression analysis of risk factors for women with PCOS and obesity

Stepwise logistic regression analysis was performed after excluding 11 variables (LH, FSH, LH/FSH, T, E2, follicle count, PSV, EDV, PI, VI, FI) to avoid overfitting and multicollinearity, with 9 candidate independent variables included. The minimum sample size was estimated to be ≥ 90 cases (α = 0.05, power = 0.80), and 211 subjects were finally enrolled to ensure stability.

**FIGURE 2:**
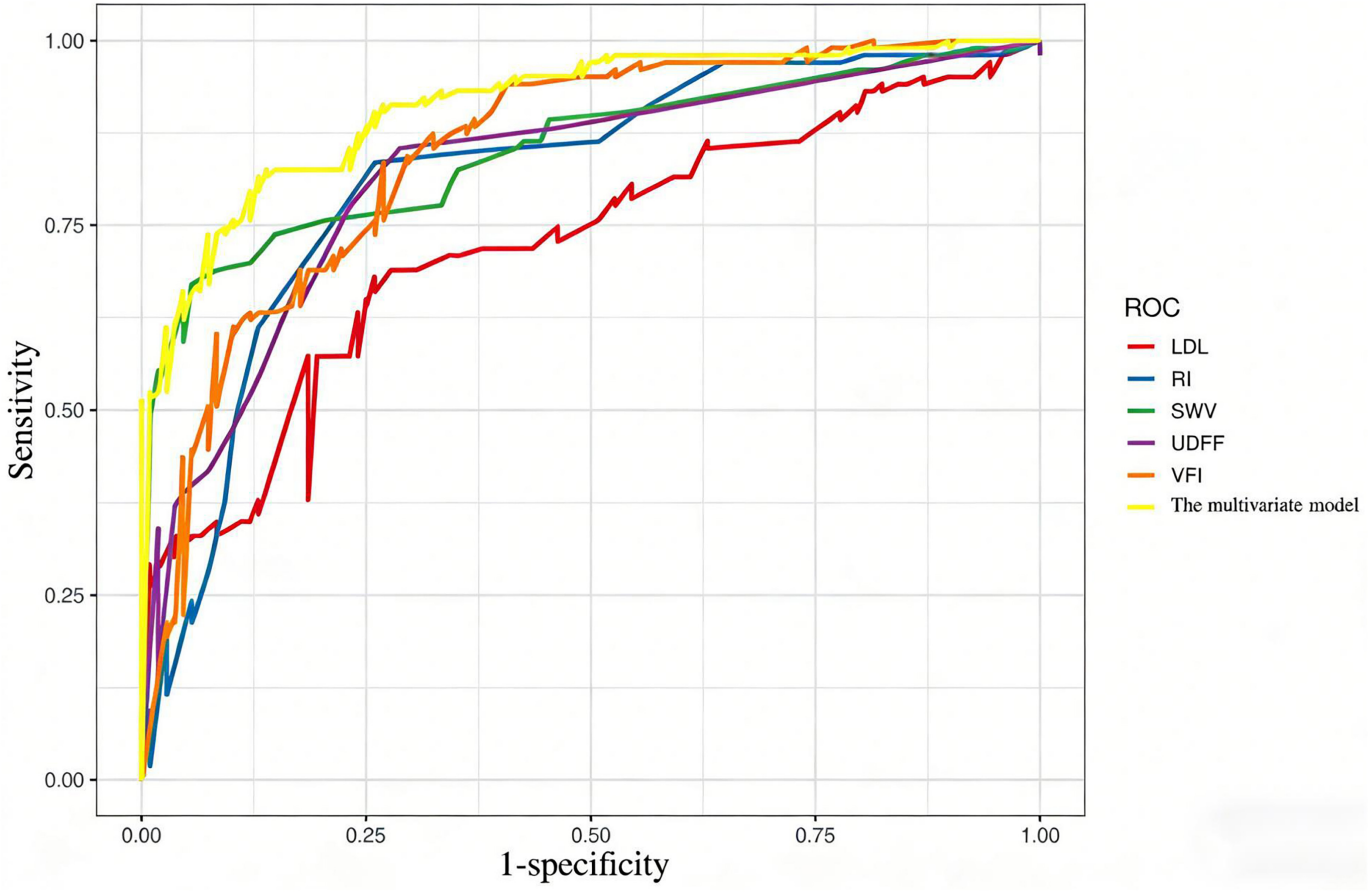
ROC curves of SWV, VFI, UDFF, RI, LDL and multivariate model for predicting PCOS in women with obesity. The multivariate model (AUC = 0.913, 95%CI: 0.872-0.954) showed better diagnostic performance than all univariate models.

SWV, VFI, UDFF, RI, and LDL were identified as independent risk factors for women with PCOS and obesity (Table 4). Multicollinearity test showed all VIF < 10 and tolerance > 0.1, indicating no severe multicollinearity (Table 5). ROC curve analysis showed the multivariate model had an AUC of 0.913 and Nagelkerke R^2^ of 0.638, indicating good fitting and strong diagnostic ability (Figure 2).

**TABLE 4:** Stepwise Logistic regression analysis of risk factors for PCOS.

Variable	Regression coefficient (β)	Standard error	OR	95%CI	*P*-value
Intercept	0.3474	0.2239	1.4153	0.9247–2.2375	0.1208
SWV	–1.667	0.3794	0.1888	0.0855–0.3822	<0.001
VFI	0.916	0.3356	2.4992	1.3268–5.0247	0.0063
UDFF	0.0915	0.4116	1.0958	0.4904–2.4994	0.8241
RI	–0.9272	0.2676	0.3956	0.2278–0.6565	<0.001
LDL	0.4619	0.3096	1.5871	0.8638–2.9325	0.1357

**TABLE 5:** Multicollinearity test of independent variables.

Variable	VIF	Toleranc	Collinearity judgment
SWV	1.881	0.532	No severe collinearity
VFI	2.564	0.39	No severe collinearity
UDFF	3.768	0.265	No severe collinearity
RI	1.583	0.632	No severe collinearity
LDL	2.122	0.471	No severe collinearity

The multivariate model (AUC = 0.913, 95%CI: 0.872–0.954) showed better diagnostic performance than all univariate models.

### 3.5 Correlation analysis between UDFF and other indices

**FIGURE 3:**
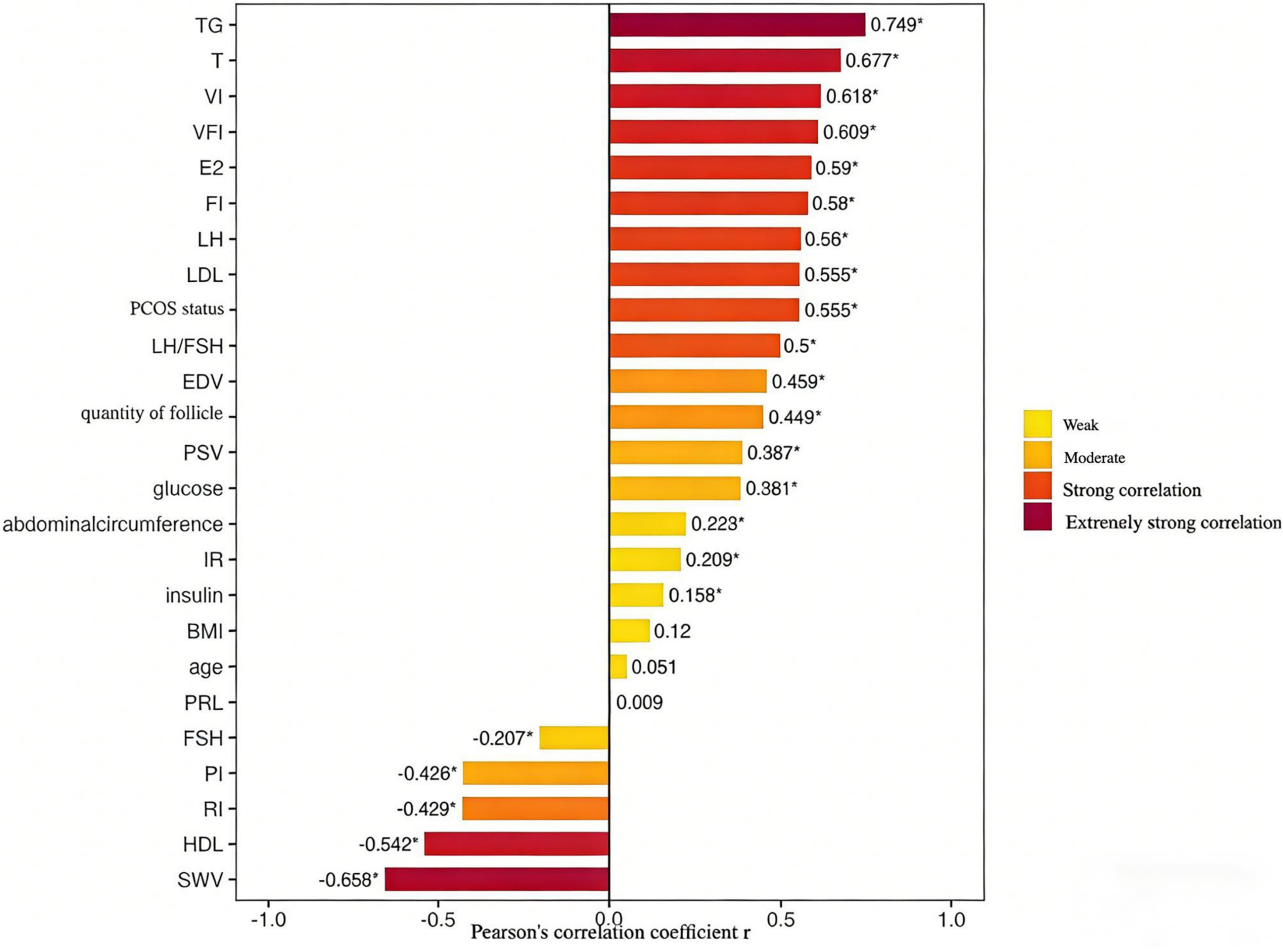
Spearman correlation coefficients between UDFF and clinical/ultrasound indices in Women with PCOS and obesity. Correlation strength: Extremely strong (| *r*| ≥ 0.7), Strong (0.5 ≤ | *r*| < 0.7), Moderate (0.3 ≤ | *r*| < 0.5), Weak (| *r*| < 0.3). *Indicates that the subsequent number is omitted.

Spearman correlation analysis revealed that UDFF was positively correlated with TG (extremely strong correlation); strongly positively correlated with T, VI, VFI, E2, FI, LH, LDL, PCOS status, and LH/FSH; and negatively correlated with SWV, HDL, RI, and PI (strong correlation). It showed a positive correlation with EDV, quantity of follicles, PSV, and glucose, and a negative correlation with RI and PI (moderate correlation) (Figure 3).

## 4 Discussion

UDFF, as a new class of ultrasound technology, is a deep-learning algorithm developed by integrating BSC and AC techniques. Its clinical application value in assessing liver fat content is gradually gaining attention (16). Relevant studies have confirmed a high consistency between UDFF and PDFF measurement results (17). Research has shown (18) that in adipose tissue samples from PCOS patients, the mRNA level of the low-density lipoprotein receptor (LDLR) is lower than in women in the control group. Since LDLR plays an important role in clearing lipoproteins containing apoB and apoE, low LDLR levels indicate a stronger lipogenic effect in PCOS patients. For PCOS patients characterized by hyperandrogenism, the activation of the IRE1α/TXNIP pathway may be involved in the occurrence of chronic inflammation in ovarian granulosa cells, thereby leading to ovarian dysfunction (19). In this study, we included an age-and BMI-matched control group to minimize the interference of obesity on the research outcomes. The results showed that compared to control group, the case group had more significant disorders in lipid metabolism, glucose metabolism, sex hormone levels, and abnormal ovarian blood flow parameters, further corroborating the association between PCOS and endocrine metabolic disorders.

The results of this study indicate that women with PCOS and obesity exhibit more pronounced abnormalities in glucose and lipid metabolism compared to the control group, which is consistent with the findings of Chen et al. (20). This suggests that polycystic ovary syndrome is associated with dysregulation across multiple metabolic pathways. In this study, no statistically significant differences were observed between the two groups in terms of fasting insulin, HDL, or PRL levels. This implies that although obesity is closely linked to insulin resistance and hyperandrogenism, and can exacerbate PCOS-related symptoms, alterations in fasting insulin, HDL, and PRL levels are not the sole pathways through which obesity influences PCOS. This finding also reflects the complexity and diversity of the pathophysiological mechanisms underlying PCOS. Therefore, in clinical management, for overweight or obese PCOS patients, greater emphasis should be placed on key aspects such as improving glucose metabolism, reducing insulin resistance, and controlling androgen levels, rather than focusing solely on regulating LH, FSH, E2, or PRL levels (21).

This study excluded 11 variables prior to conducting stepwise logistic regression for the following reasons: the diagnostic criteria for polycystic ovary syndrome include sex hormone indicators, which could lead to model overfitting; furthermore, variables exhibiting extremely high multicollinearity, such as VFI, VI, FI, EDV, PSV, RI, and PI, were addressed by removing VI, FI, EDV, PSV, and PI, while retaining VFI and RI, which hold greater clinical significance, for inclusion in the model (22). Among the variables ultimately incorporated into the model, the ultrasound indicators demonstrated the highest AUC values. This finding highlights the strong diagnostic capability of novel ultrasound techniques for polycystic ovary syndrome.

Levels of VI, VFI, FI, EDV, and PSV were higher in women with PCOS and obesity compared to the simple obesity group, while RI and PI were lower than in the control group. A potential explanation is that the presence of numerous luteinized granulosa cells in PCOS patients leads to excessive secretion of vascular endothelial growth factor, increased vascular wall permeability, coupled with persistent high levels of LH stimulating ovarian stromal vascular dilation, which may result in decreased ovarian RI and abnormally increased blood flow (8, 23, 24). The inclusion of VFI in the regression model underscores the strong diagnostic utility of ovarian ultrasound hemodynamics for PCOS.

In this study, UDFF, SWV, and VFI all demonstrated good diagnostic performance, with AUC values exceeding 0.80. UDFF effectively quantifies liver fat content, SWV provides a non-invasive assessment of liver stiffness, and VFI quantifies ovarian stromal blood flow. The combination of these indicators may offer a more comprehensive evaluation tool for clinical practice.

In the correlation analysis between UDFF and various indicators, a very strong correlation with TG was observed, reflecting UDFF’s fundamental role in quantitatively assessing liver fat content—a finding consistent with the results of Verdan et al. (25). Strong correlations were also found with SWV, HDL, LDL, VI, FI, VFI, T, LH, LH/FSH, and E2, indicating that UDFF can perform risk stratification for obese PCOS patients. Individuals with higher UDFF values exhibit a greater probability of having PCOS, more severe metabolic abnormalities, higher ovarian stromal hemodynamic indicators, and consequently, more severe clinical manifestations. This suggests that UDFF not only reflects the degree of fat accumulation but is also indirectly associated with the pathophysiological alterations of the disease (26). This implies that UDFF can help identify populations at high risk for disease progression, facilitating early intervention to prevent advancement to more severe disease states, particularly demonstrating significant potential in preventing liver cirrhosis. As a non-invasive, convenient, and reproducible quantitative indicator, UDFF can, to some extent, reflect disorders in glucose and lipid metabolism, endocrine imbalances, and ovarian morphological and hemodynamic parameters. It may serve as an indicator for metabolic risk assessment in women with PCOS and obesity, although its clinical translation and application require integrated judgment combined with multi-dimensional indicators.

The strength of this study lies in its maximal reduction of obesity-related confounding through BMI adjustment, combined multi-dimensional analysis of novel ultrasound indicators such as UDFF, SWV, and VFI with glucolipid metabolism, sex hormones, and ovarian blood flow parameters, thereby confirming their value in the diagnosis and condition assessment of women with PCOS and obesity. By constructing a diagnostic model that combines ultrasound and clinical indicators, it provides a non-invasive and convenient assessment tool for clinical practice, balancing scientific rigor with clinical utility. This study also has several limitations: First, residual confounding factors could not be completely excluded, including diet, physical activity, and medication history beyond hormone-related drugs. Second, the single-center design with clinic-based recruitment may lead to potential selection bias, which may limit the generalizability of the results. Furthermore, subgroup analyses were not performed, making it difficult to comprehensively verify the applicable scope and grading criteria for UDFF. Therefore, the conclusions still require further validation through multicenter, large-sample prospective studies.

## 5 Conclusion

In summary, UDFF demonstrates promising application prospects in the evaluation of women with PCOS and obesity. Through an in-depth analysis in this study, we have provided robust scientific evidence supporting the use of UDFF as a screening tool for health management in this population. The findings contribute to a deeper understanding of glucolipid metabolism disorders, sex hormone imbalances, and various ovarian-related ultrasound indicators in PCOS patients. Furthermore, this advancement may help reduce the reliance on invasive testing methods. Future large-scale, multicenter, prospective studies are warranted to further validate these results and promote the adoption of this technology as a non-invasive assessment tool for Women with PCOS and obesity.

## Data Availability

The raw data supporting the conclusions of this article will be made available by the authors, without undue reservation.
